# Supplementary figures and images for: Particle-based simulations reveal two positive feedback loops allow relocation and stabilization of the polarity site during yeast mating
Source: PLoS Comput Biol. 2023 Oct 2;19(10):e1011523. doi: 10.1371/journal.pcbi.1011523 (PMC10569529; doi:10.1371/journal.pcbi.1011523)

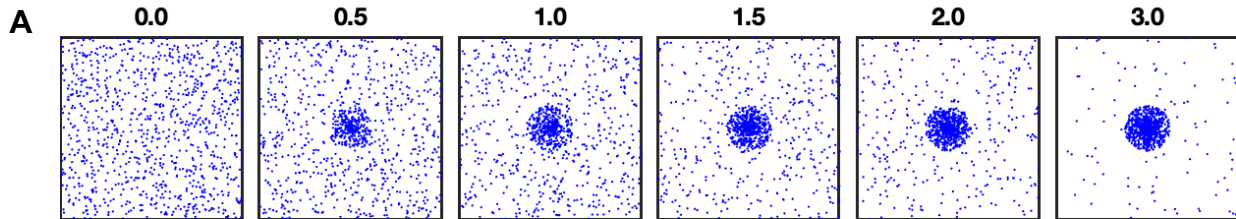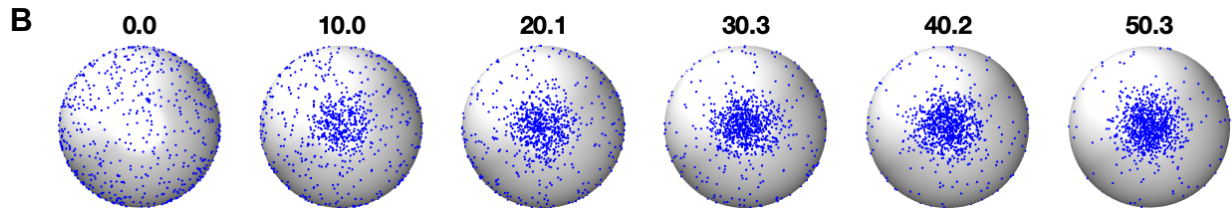

Supplement: S1 Fig — Cluster distributions corresponding to the listed K values. A) 2D examples for a square domain of length 8.8623 μm. B) 3D examples for a sphere of radius 2.5 μm. (PDF) [file pcbi.1011523.s001.pdf]

**A**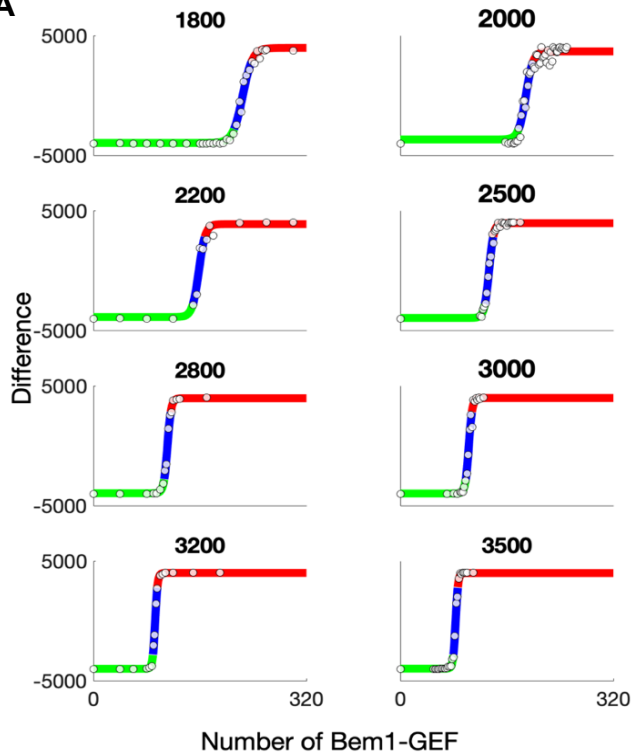**B**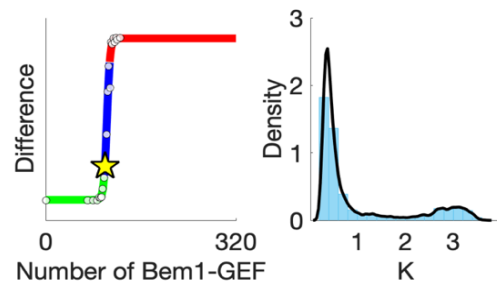**C**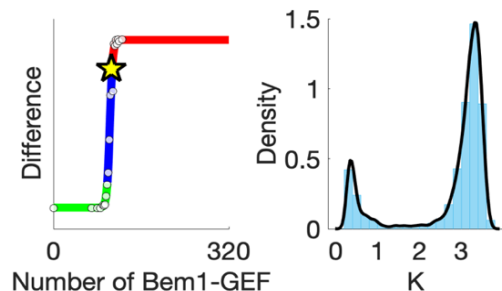

Supplement: S2 Fig — A) Difference in the number of polarized and unpolarized states as a function of Bem1-GEF abundance. Data points (o) represent simulation results for the total number of Cdc42 molecules indicated in the plot title. Curves represent fits to the data using a logistic function (unpolarized regime—green, polarized regime—red, and transient regime—blue). B and C) Distributions for K in the transient regime using a total Cdc42 abundance of 3000. Stars indicate the Bem1-GEF abundances used to produce the distributions (100 lower panels and 106 upper panels). (PDF) [file pcbi.1011523.s002.pdf]

**A****Ripley's K-function**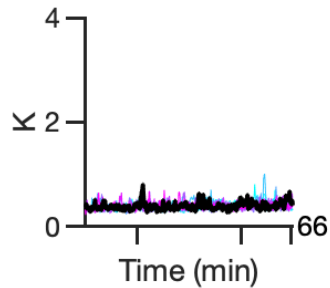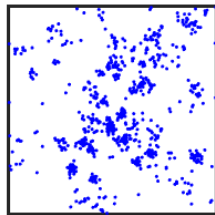

17 min

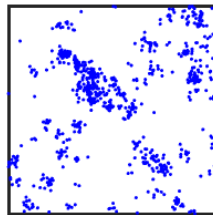

50 min

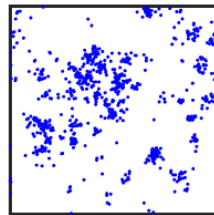

66 min

**B**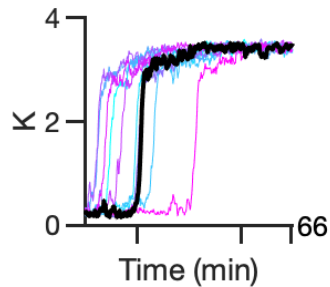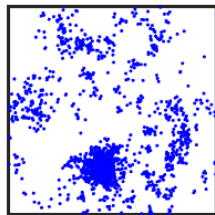

17 min

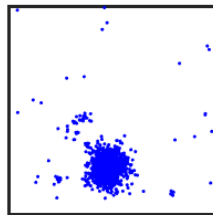

50 min

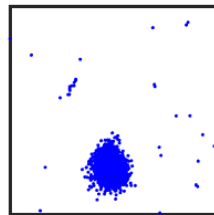

66 min

Supplement: S3 Fig — A) Time series for K for 10 simulations in the unpolarized regime (left panel). Distributions for active Cdc42 taken form black time series using time points indicated with ticks on the time axis (right panels). Simulations were performed using uniform random distributions of molecules as initial conditions with 3000 Cdc42 and 70 Bem1-GEF molecules. B) Same as A except with 3000 Cdc42 and 120 Bem1-GEF molecules. (PDF) [file pcbi.1011523.s003.pdf]

**A**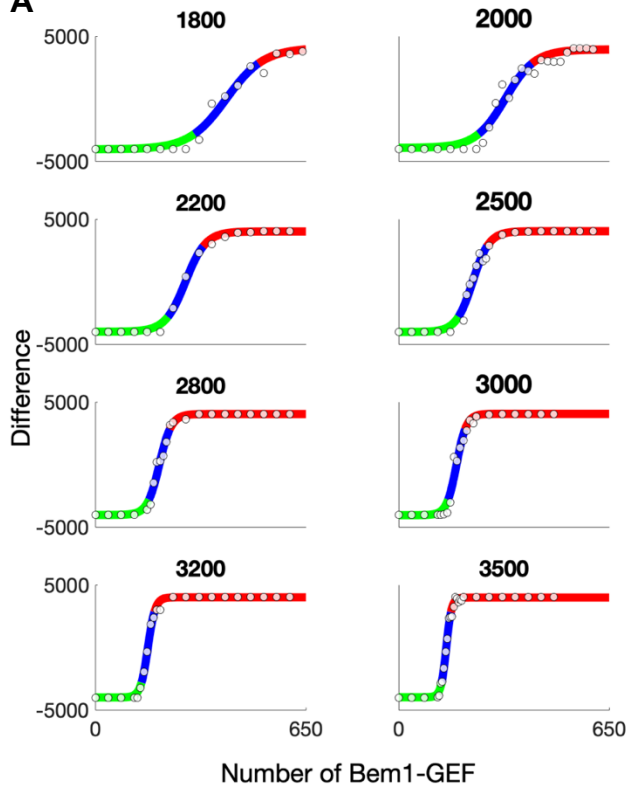**B**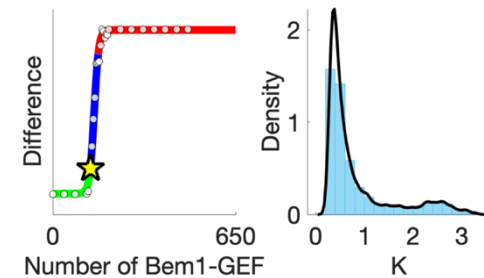**C**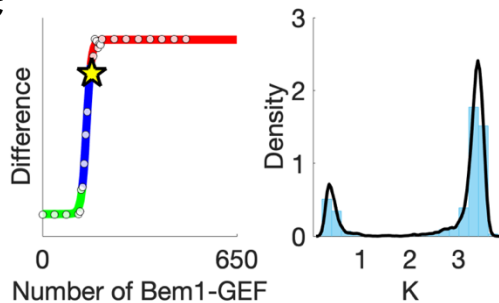

Supplement: S4 Fig — A) Difference in the number of polarized and unpolarized states as a function of Bem1-GEF abundance. Data points (o) represent simulation results for the total number of Cdc42 molecules indicated in the plot title. Curves represent fits to the data using a logistic function (unpolarized regime—green, polarized regime—red, and transient regime—blue). B and C) Distributions for K in the transient regime using a total Cdc42 abundance of 3500. Stars indicate the Bem1-GEF abundances (135 lower panels and 165 upper panels). All simulations were performed using 30 Far1-GEF and 2500 receptor molecules. (PDF) [file pcbi.1011523.s004.pdf]

# A

## Ripley's K-function

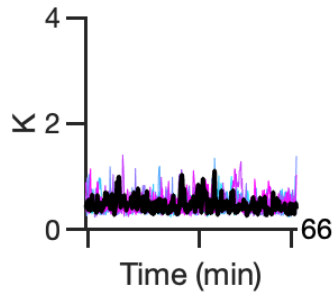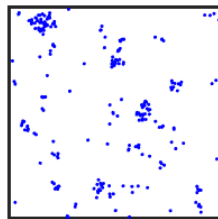

1 min

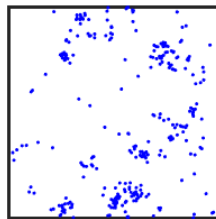

36 min

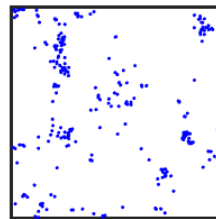

65 min

# B

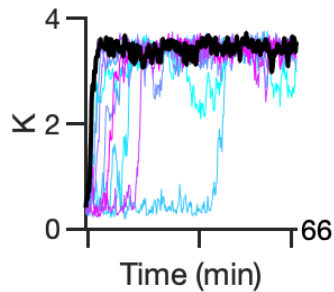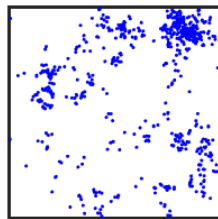

1 min

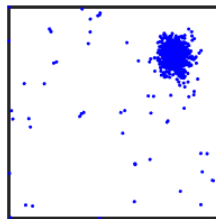

36 min

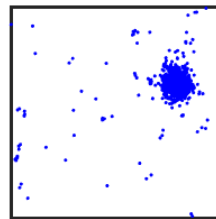

65 min

Supplement: S5 Fig — A) Time series for K for 10 simulations in the unpolarized regime (left panel). Distributions for active Cdc42 taken form black time series using time points indicated with ticks on the time axis (right panels). Simulations were performed using uniform random distributions of molecules as initial conditions with 3000 Cdc42, 80 Bem1-GEF, 30 Far1-GEF and 2500 receptor molecules. B) Same as A except with 280 Bem1-GEF. (PDF) [file pcbi.1011523.s005.pdf]

A

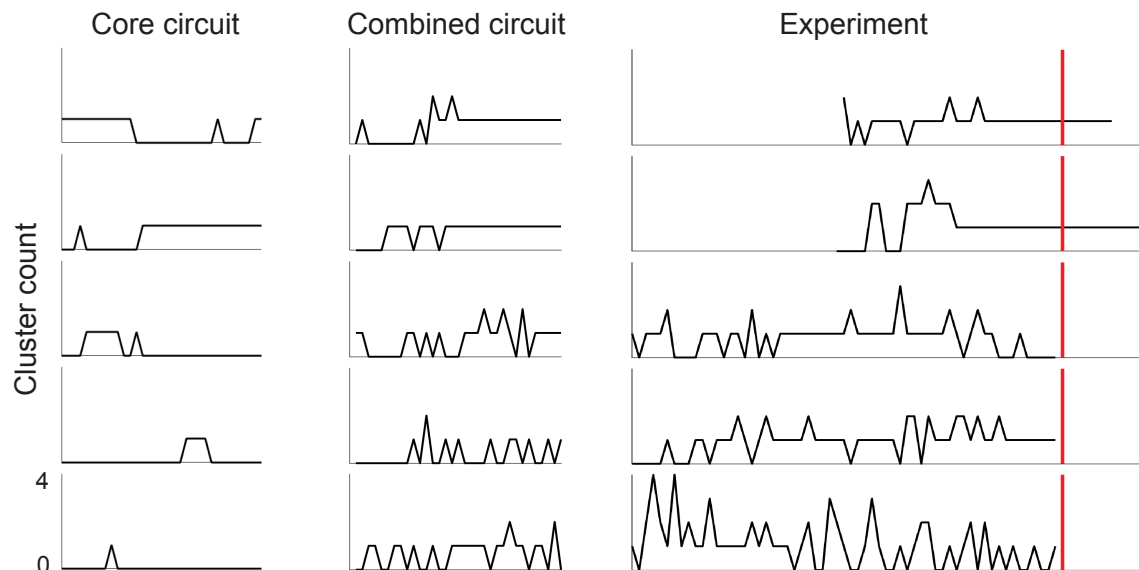

B

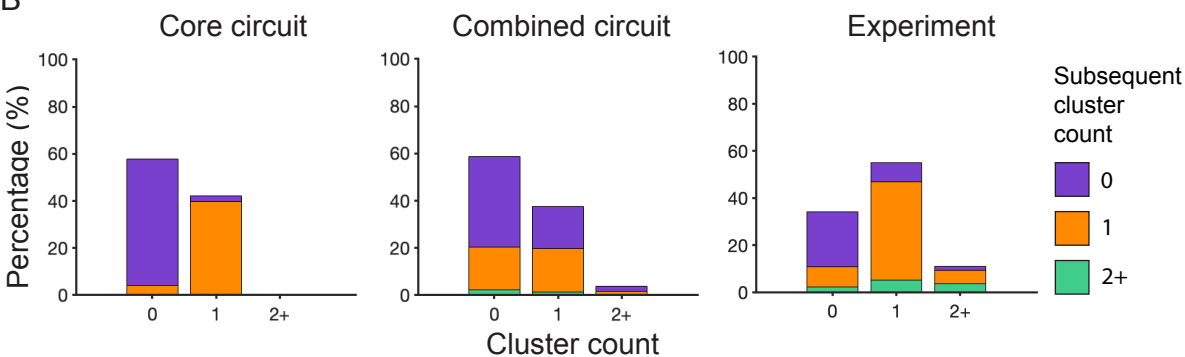

C

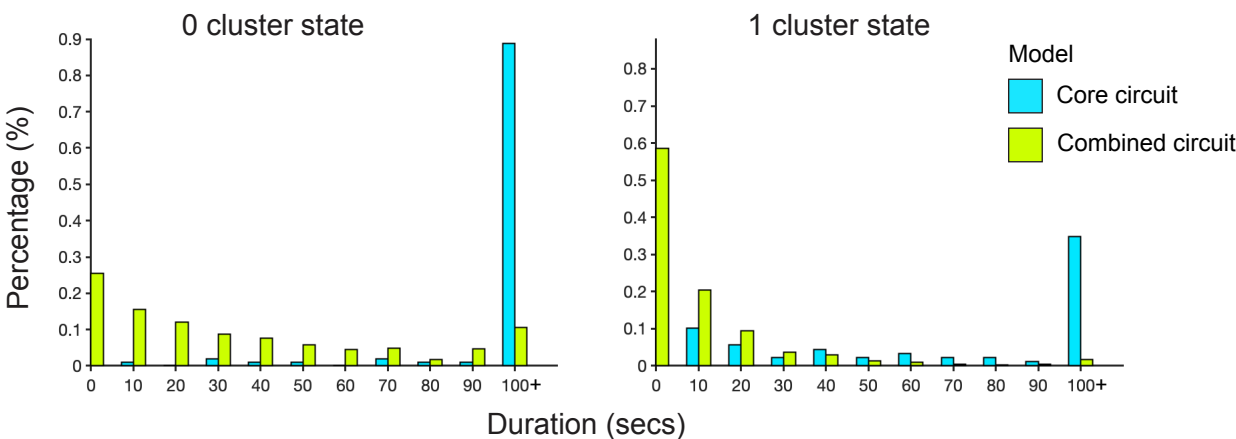

Supplement: S6 Fig — A) Five examples of time series for the number of clusters, B) the distribution of cluster counts, the transition probabilities to a subsequent cluster count, and C) the dwell time of clusters for simulations of the core polarity circuit with 3000 Cdc42 and 102 Bem1-GEF molecules and simulations of the combined polarity circuit with 3000 Cdc42 and 170 Bem1-GEF molecules. Note that the images were taken at 120-second intervals in experiments, so we were not able to compare dwell times of clusters between simulations and experiments. The total time duration in A) are 4000 secs with intervals of 120 secs. The red line denotes the time at which cells became committed. (PDF) [file pcbi.1011523.s006.pdf]

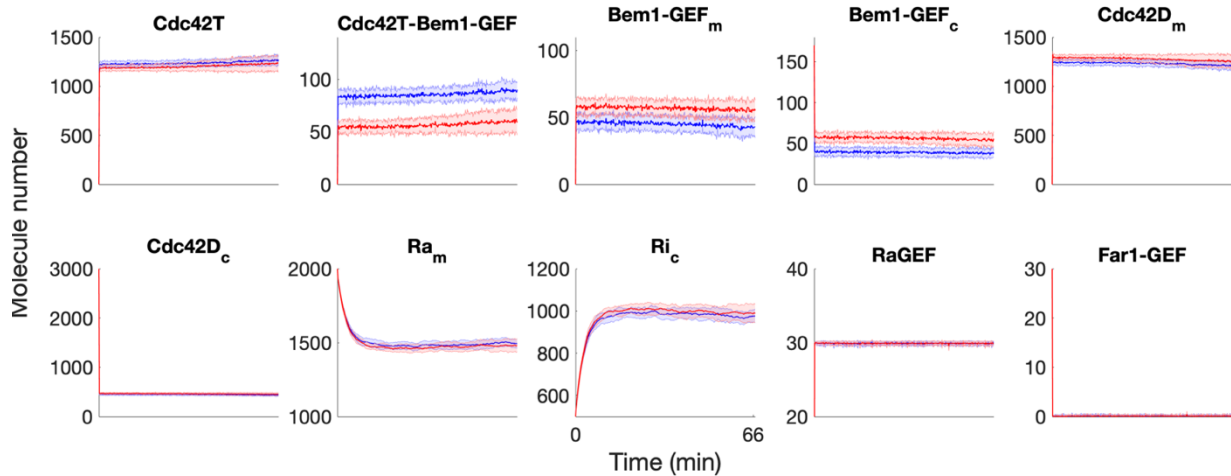

Supplement: S7 Fig — Simulations were performed with 3000 Cdc42, 170 Bem1-GEF, 30 Far1-GEF, and 2500 receptor molecules. Simulations started with uniformly distributed molecules. Lines represent molecule number averages and the shaded areas represent ± std for 30 simulations (3D –blue, 2D –red). All simulations were run for 66 min. (PDF) [file pcbi.1011523.s007.pdf]

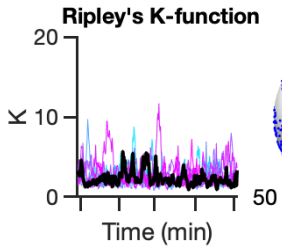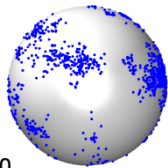

1 min

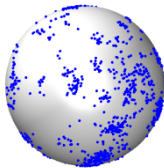

13 min

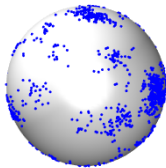

24 min

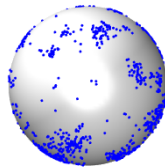

37 min

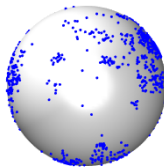

49 min

Supplement: S8 Fig — Simulations were performed using 3000 Cdc42, 170 Bem1-GEF, 2500 receptor, 30 Far1-GEF and 1.5 nM uniform concentration of pheromone molecules. (PDF) [file pcbi.1011523.s008.pdf]
